# Amino Acid Composition in Different Tissues of Iceland Scallop from the Barents Sea

**DOI:** 10.3390/ani14020230

**Published:** 2024-01-11

**Authors:** Alexander G. Dvoretsky, Ekaterina D. Obluchinskaya, Elena V. Gorshenina, Vladimir G. Dvoretsky

**Affiliations:** Murmansk Marine Biological Institute of the Russian Academy of Sciences (MMBI RAS), 183038 Murmansk, Russia

**Keywords:** *Chlamys islandica*, Barents Sea, amino acids, muscle, gonad, mantle

## Abstract

**Simple Summary:**

The adductor muscles of scallops are highly prized for their exceptional taste and nutritional value. Additionally, by-products of scallops show potential for the production of functional foods, animal feed, and nutraceuticals. To address the paucity of information regarding the amino acid composition of Iceland scallops from the coastal Barents Sea, we conducted biochemical analyses of various tissues from this species. Our findings revealed the highest contents of glycine and arginine, two amino acids widely implicated in the physiological processes of scallops worldwide. The consistency of amino acid composition across the muscle, gonad, and mantle tissues, as well as their lack of differentiation between male and female scallops, was noteworthy. A negative correlation was observed between the gonad index and amino acid contents in the muscle, while a positive relationship was found between muscle amino acid content and depth. Furthermore, amino acid contents in the mantle demonstrated a positive correlation with water temperature. Scallop by-products hold significant promise for the food and pharmaceutical industries.

**Abstract:**

The Iceland scallop from the Barents Sea is a commercially important species with promising aquaculture potential, but information on the biochemical properties of its tissues is limited. Our analysis of the adductor muscle, gonad, and mantle of this bivalve mollusk from coastal waters provided insight into its amino acid composition. Biochemical analysis revealed predominant levels of glycine (11.8, 11.5, and 9.6 mg g^−1^, respectively) and arginine (11.2, 8.3, and 5.8 mg g^−1^, respectively). While multivariate comparisons did not reveal significant differences in amino acid composition between the tissues, single comparisons showed significantly higher levels of arginine and leucine in the adductor muscle compared to those of the mantle. The abundant presence of glycine and arginine underscores their importance in maintaining basic physiological processes, consistent with other scallop species. Redundancy analysis identified water depth and scallop gonad index as influential factors shaping the amino acid profile in the adductor muscle. In the case of the mantle, water temperature emerged as the main driver of amino acid content. Our results confirm the richness of essential amino acids in scallop by-products and highlight their potential for human consumption, production of feed ingredients for farmed animals, and nutraceuticals.

## 1. Introduction

The Barents Sea represents a unique high-latitude Large Marine Ecosystem owing to its high primary productivity and biodiversity [1,2,3] arising from interactions between water masses of disparate origin, namely cold Arctic waters and relatively warmer Arctic waters [4,5,6,7]. This ecosystem sustains major fisheries primarily targeting cod (*Gadus morhua*), haddock (*Melanogrammus aeglefinus*), saithe (*Pollachius virens*), and capelin (*Mallotus villosus*). Crustacean targeted fisheries predominantly focus on northern shrimp (*Pandalus borealis*), red king crab (*Paralithodes camtschaticus*), and snow crab (*Chionoecetes opilio*) [6,8,9]. The latter two species are invasive yet well adapted to their novel environments [10,11]. Coastal regions of the sea also exhibit high productivity and richness in terms of biota, possessing great potential for small-scale fisheries and aquaculture, particularly in the context of shellfish [12,13]. Bivalve mollusks significantly contribute to inshore benthic communities and comprise a multitude of species [14,15]. Of these, scallops are often considered most promising in terms of potential for fishery exploitation and aquaculture [16,17]. Scallops are also considered reliable biosensors of environmental conditions [18]. The commercial success of pectinid fisheries and aquaculture is attributable to the global appreciation of the gastronomic qualities of the striated adductor muscle of these bivalves. For this reason alone, investigation of these muscles is merited. Bivalve meats extracted from the shell comprise the majority of pectinid products consumed globally, yet only constitute approximately 10% of the total mass of landed individuals [19]. Scallop byproducts include the mantle, gonad, gill, liver and kidney tissues, among which the mantle and gonads are considered to have the greatest total mass and potential commercial applications [20].

The Iceland scallop, *Chlamys islandica*, is the most abundant scallop species found in the Barents Sea. This medium-sized mollusk exhibits a wide sub-polar distribution, ranging from Massachusetts on the east coast of the USA to Greenland, Iceland, Norway, and extending as far east as the Kara Sea [21]. Along the coast, it is typically found on the inner sides of fjords with shallow sills and low bottom temperatures ranging from 9 °C to 21 °C [22]. The species is found at depths ranging from 10 to 250 m, with the highest abundance observed at depths shallower than 100 m. *Chlamys islandica* displays a preference for hard coarse sediments, comprised of sand, gravel, shells, occasionally intermixed with clay and fine sand [22]. Such areas often coincide with strong ocean currents, leading to scallops being ubiquitously attached to stones or shells. Sexual maturity is achieved between 2.5 and 6 years depending on environmental conditions [21].

Large-scale fisheries for Iceland scallops are currently not present in the Barents Sea. However, in the past few decades, commercial dredging has specifically targeted this species, with the most profitable fishing zones situated near Cape Svyatoy Nos. From 1995 to 2001, the total annual catch in this region increased considerably to reach a peak of 10–13 thousand metric tons [17]. Despite the lack of large-scale commercial fishing, the scallop continues to attract sport divers at specific coastal sites and has considerable potential for commercial aquaculture [16].

Due to their economic significance, Iceland scallops have undergone extensive research in the Barents Sea, resulting in a complicated understanding of many aspects of their life cycle. Population dynamics, patterns of reproduction, growth rates, feeding habits, and mortality rates have all received detailed attention [17,23,24]. Furthermore, particular aspects of technological processing have also been examined [17]. However, some gaps in knowledge persist, particularly regarding the biochemical composition of the scallop flesh and by-products. Specifically, there is a lack of data on the amino acid composition of *Chlamys islandica* tissues and factors affecting this composition. Recent research has highlighted the potential metabolic benefits of scallop flesh, such as its ability to prevent diet-induced obesity [25,26]. Consequently, understanding the amino acid composition is crucial for the food, medical, and pharmaceutical industries.

Therefore, our aim was to determine the content of 16 proteinogenic amino acids in the adductor muscle, mantle and gonads of *Chlamys islandica* inhabiting the coastal areas of the Barents Sea, and to evaluate the role of various factors in shaping the amino acid levels in these tissues.

## 2. Materials and Methods

### 2.1. Study Area

**Figure 1:**
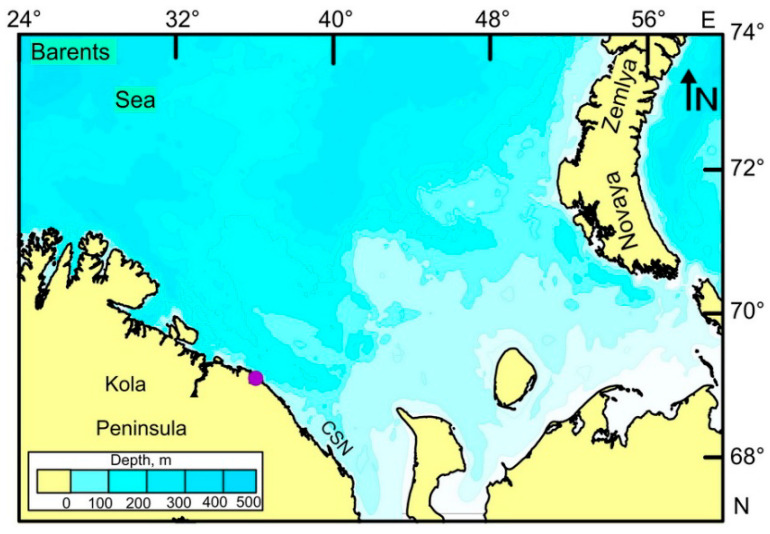
Map denoting the location of the study area, Dalnezelenetskaya Bay (indicated by the violet circle), and the sites of abundant scallop beds in proximity to Cape Svyatoy Nos (CSN) in the Barents Sea, Russia.

The study was carried out in Dalnezelenetskaya Bay, which is a semi-enclosed gulf situated on the eastern coast of the Kola Peninsula, Barents Sea, Russia (Figure 1).

This small bay has approximately equal width and length of 2 km each, encompassing a total area of 2.23 km^2^ [27]. A system of 5 minor islands separates the bay from the open sea. Dalnezelenetskaya Bay is mostly a shallow marine basin, with an average depth of 7 m. However, its western sector features a maximum depth of 50–55 m. The tidal stream intensity in the bay is typically low, with velocities rarely exceeding 1 km h^−1^. The bay demonstrates significant tidal amplitude levels of 4–4.1 m, guaranteeing a consistent exchange of water between the bay’s inner regions and the open sea. The surface water temperature in Dalnezelenetskaya Bay varies throughout the year, ranging from 0.7 °C in February and March to 9.7 °C in August. The bay also demonstrates relatively weak fluctuations in salinity, with a minimum value of 32.2 psu in May due to melting processes and a maximum value of 34.3 psu in December and January [27]. Dissolved oxygen varies between 94% saturation in December and 124% in May [12].

### 2.2. Sampling and Processing

Benthic underwater surveys were carried out in July of both 2022 and 2023 at depths ranging from 15 to 33 m. During the surveys, Iceland scallops were collected along standard transects within the bay. The sediment type (hard bottom, soft bottom, or mixed sediments), depth, and water temperature were recorded at each transect. The scallops, which were collected using SCUBA diving techniques, were promptly transported to the nearby coastal laboratory for further analysis.

Due to heavy epibiotic fouling, each scallop shell was preliminarily cleared of attached organisms using a scalpel. Following this, the scallops were measured for shell length (SL, mm) using calibrated calipers [17]. Digital precision weighing was then employed to determine the total wet weight of each mollusk. Through a mid-dorsoventral incision of the adductor muscle using a scalpel, the internal organs including the gonad, the mantle, and the adductor muscle were carefully dissected and separated from one another. Wet weights of these dissected tissues were subsequently determined using digital precision scales. As all mollusks exhibited mature development, sex was assessed according to gonad coloration, white indicating male and orange signifying female. The muscle index (MI = 100 × Muscle wet weight/Total body wet weight), the gonad index (GI = 100 × Gonad wet weight/Total body wet weight), and the mantle index (MNI = 100 × Mantle wet weight/Total body wet weight) were also calculated.

To perform amino acid analyses, the collected samples were frozen and transported to the biochemical laboratory at the Murmansk Marine Biological Institute (Murmansk, Russia).

### 2.3. Biochemical Assay

According to the method for determining the mass fraction of amino acids in foods, feed, and raw materials (M-02-902-142-07, Analyt Ltd., Saint Petersburg, Russia), the following steps were conducted:

(1) Each sample was mechanically homogenized using a universal homogenizer (Ultra-Turrax Tube Driv, IKA, IKA-Werke GmbH & Co. KG, Staufen, Germany) equipped with DT-20-M-gamma tubes at 6000 rpm, and approximately 100 mg of the sample was transferred to a vial for further analysis.
(2) The sample was then hydrolyzed in the nitrogen-filled vial using 10 mL of 6 M HCl at a temperature of 110 °C for a duration of 24 h.
(3) After the hydrolysis process, the sample was cooled to room temperature, followed by filtration through a membrane filter of 2 µm and mixing.
(4) A 0.1 mL aliquot of the sample was pipetted and evaporated under a hot plate at 65 °C.
(5) The evaporated aliquot was then dissolved in a mixture comprising 0.1 mL of a 0.15 M L^−1^ NaOH solution, 0.35 mL of a solution of phenylisothiocyanate in isopropanol, and 0.05 mL of distilled water.
(6) The resulting mixture was stored for 20 min and evaporated until complete drying.
(7) The dried sample was dissolved in 1 mL of distilled water and centrifuged at 10,000 r.p.m. for 5 min.
(8) Finally, the resulting sample was analyzed using a Shimadzu LC-20AD Prominence high-performance liquid chromatography system equipped with a detector Shimadzu SPD-M20A Prominence (Shimadzu, Japan) and a 250 mm × 4.6 mm × 5 µm column Supelco C18 (Supelco, Bellefonte, PA, USA).

This method enables detection of 16 amino acids (exceptions include tryptophan, methionine, and cystine + cysteine). Results are reported as mg of amino acid per 1 g of tissue wet weight.

### 2.4. Statistical Analysis

One-way analysis of variance (ANOVA) was used to test for differences in biometric data between males and females because these data met the required assumptions for parametric tests. Principal component analysis (PCA) was used to identify underlying patterns in amino acid content to facilitate data simplification and interpretation. In addition, two-way permutational multivariate analysis of variance (PERMANOVA) with 9999 permutations using the Bray–Curtis dissimilarity index on the raw data was performed to explore differences in amino acid composition among the three scallop tissues (adductor muscle, mantle, and gonad) and between male and female specimens. In addition, individual amino acid levels were examined for potential differences using ANOVA with Tukey’s multiple comparison tests for data conforming to normal distribution, which was assessed using the Shapiro–Wilk test and a modified version of Levene’s test for normal distribution and homoscedasticity of data. If these assumptions were not met, the data were transformed. SIMPER (similarity percentages) analysis was performed to determine the contribution of each amino acid to the average dissimilarity between groups.

Redundancy analysis (RDA), a direct gradient analysis of amino acid data, was applied to identify pertinent variables that best explain the amino acid content in Iceland scallops. The independent variable matrix included ranked sediment types (hard bottom = 1, mixed sediments = 2, and soft bottom = 3), water temperature, depth, SL, MI, GI, and MNI. Prior to analysis, amino acid contents were log_10_-transformed. A Monte Carlo permutation test (n = 999) was conducted to identify the factors that most effectively explain the distribution of the dependent variables, with environmental variables screened for collinearity to exclude those with a variance inflation factor greater than 10 (VIF > 10). RDA was applied separately to each tissue type.

Statistical analyses were performed utilizing NCSS PASS 2004 software for ANOVA, PAST 3.26 software for PERMANOVA and SIMPER, and CANOCO 4.5 software for RDA.

## 3. Results

### 3.1. Scallops

A total of 13 scallops, including 3 from the 2022 survey and 10 from the 2023 survey, were collected within the study area, comprising 6 males and 7 females. Their size and weight characteristics, together with the calculated indices, are summarized in Table 1.

ANOVA revealed a significant difference between the sexes for mantle weight (F = 4.91, *p* = 0.049) and MNI (F = 8.42, *p* = 0.015), both of which were higher in males.

### 3.2. Amino Acid Composition

Of the 16 amino acids detected, the highest contents were found for glycine and arginine, as shown in Table 2.

**Figure 2:**
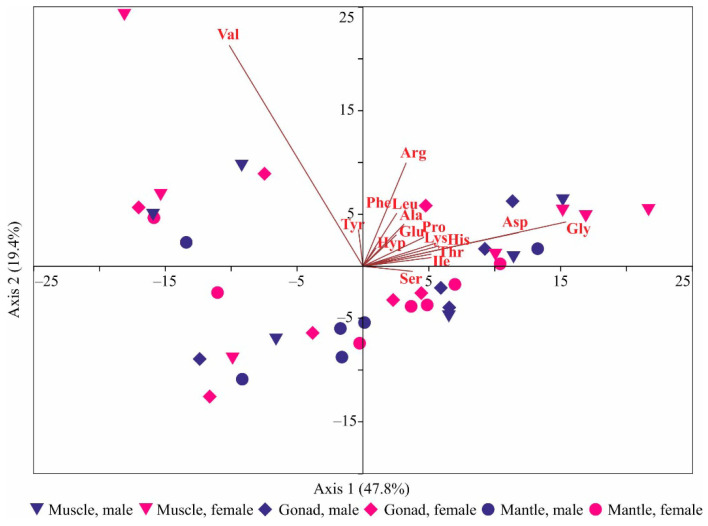
Biplot of principal component analysis based on amino acid contents in tissues of Iceland scallop from the Barents Sea. Asp—aspartic acid, Glu—glutamic acid, Hyp—Hydroxyproline, Ser—serine, Gly—glycine, His—histidine, Arg—arginine, Thr—threonine, Ala—alanine, Pro—proline, Tyr—tyrosine, Val—valine, Ile—isoleucine, Leu—leucine, Phe—phenylalanine, Lys—lysine.

PCA revealed that the first two principal components, with eigenvalues > 1, accounted for 60.2% of the total variance. Scallops were primarily separated along Axis 1 based on glycine and asparagine content, with samples rich in these amino acids generally exhibiting positive PC1 scores and those with lower contents showing negative PC1 scores (Figure 2).

The second axis primarily segregated the scallops according to valine and arginine content. Samples with higher contents had positive PC2 scores, while those with lower contents had negative PC2 scores (Figure 2). Notably, there was no clear visual separation between different tissue types or sexes. Two-way PERMANOVA also indicated no significant differences among the three tissue types (F = 1.33, *p* = 0.218) and between male and female scallops (F = 0.53, *p* = 0.736). ANOVA generally confirmed these results, with two exceptions: arginine (F = 7.87, *p* = 0.002) and leucine (F = 4.09, *p* = 0.025), for which muscle contents were significantly higher compared to mantle contents. SIMPER analysis revealed the greatest dissimilarity between muscles and mantles, while the lowest dissimilarity was observed for the gonad–mantle pair (Table 3). Glycine and valine were identified as the most significant contributors to the dissimilarity between different tissues (Table 3).

### 3.3. Factors

**Figure 3:**
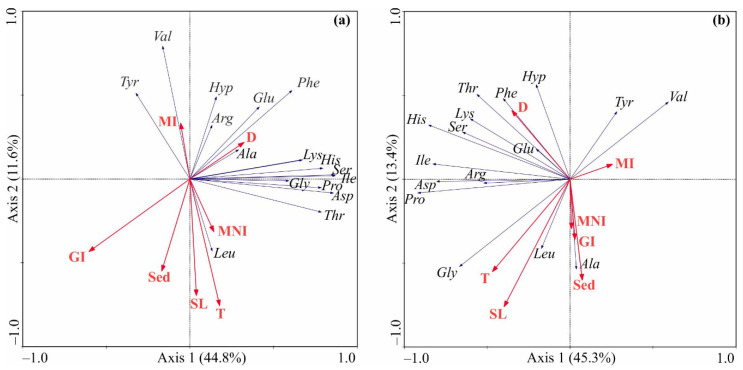
Ordination biplot of redundancy analysis based on amino acid composition in the scallop adductor muscle (**a**) and mantle (**b**) and their relations to environmental variables in Dalnezelenetskaya Bay. The proportions of the total variability explained by the first two axes are given. Independent variables: D—depth, T—water temperature, SL—shell length, Sed—sediments, GI—gonad index, MI—muscle index, MNI—mantle index. Dependent variables: Asp—aspartic acid, Glu—glutamic acid, Hyp—hydroxyproline, Ser—serine, Gly—glycine, His—histidine, Arg—arginine, Thr—threonine, Ala—alanine, Pro—proline, Tyr—tyrosine, Val—valine, Ile—isoleucine, Leu—leucine, Phe—phenylalanine, Lys—lysine.

Regarding the RDA based on the amino acid content in muscles, the first axis accounted for 56.4% of total variation. Water depth demonstrated a positive correlation with Axis 1 and the majority of amino acids, while strong negative correlations with both axes were found for the gonad index. The leucine content showed a negative correlation with the second axis (Figure 3a).

A forward selection procedure revealed that the most significant factors explaining the variance in amino acid content in the muscle were the gonad index and depth (Table 4). The former factor exhibited a negative influence on amino acid content, while the latter promoted higher contents of amino acids in the scallop muscle.

The RDA model calculated for the gonad data showed no significant associations between input and output variables.

In the case of mantle data, the RDA model was significant, with the first two axes explaining 58.7% of the total variation. Axis 1 was negatively correlated with depth and water temperature, and 13 of the 16 amino acids followed the same pattern (Figure 3b). Axis 2 was positively correlated with depth and negatively with water temperature and SL. Strong negative associations were found for this axis and glycine, leucine, and alanine. The Monte Carlo permutation test indicated that only water temperature significantly contributed to the RDA model (Table 4), having a positive effect on glycine and leucine contents.

## 4. Discussion

### 4.1. Scallops

All scallops collected in Dalnezelenetskaya Bay had a SL greater than 70 mm, reflecting their age exceeding 8 years and reaching sexual maturity [17]. Most specimens were larger than 80 mm, a criterion established for commercial individuals. Previous research has shown that the highest variability in weight occurs in the gonad, with maximum values recorded prior to spawning in the spring period [17]. In our study, the mean gonad weight was 14.0 g in males and 11.8 g in females. Comparatively, in other parts of the Barents Sea, pre-spawning gonad weights reach 10 g in males and 15 g in females. The GI in our scallops was lower than in mollusks from scallop beds near Cape Svyatoy Nos, where July values ranged from 8.8 to 16.7% in females and from 9.7 to 12.7% in males [17]. These differences likely reflect less favorable habitat conditions in Dalnezelenetskaya Bay, where scallops have an irregular distribution, as opposed to the high-density population at Cape Svyatoy Nos.

Muscle weight also exhibits seasonal variations, with minimal values during the pre-spawning period when valuable substances are allocated to the gonads to maintain reproductive processes [17]. In July, there is a recovery of muscle weight, and our scallops exhibited intermediate levels of this parameter. Our data are comparable to those recorded in Cape Svyatoy Nos, where the MI in July ranged from 10.4% to 15.6% in females and from 11.7% to 17.3% in males [17].

Distinct disparities in mantle weight and MNI were noted in relation to the sex of the scallops. The mantle, a bifacial epithelial membrane, engages in an array of crucial biological activities, including shell and ligament secretion, sensory reception, and maneuvers aiding in locomotion through the use of the velum [28]. The elevated mantle weights and MNI documented in males may be indicative of sex-specific behavioral divergences or differences in the functional demands placed on this tissue, such as increased locomotor ability or secretion activity.

### 4.2. Amino Acid Composition

In the three tissues analyzed, glycine and arginine were identified as the dominant amino acids. Similar predominance of these amino acids, particularly glycine, has been documented in the adductor muscle of several scallop species including the Pacific lions-paw scallop (*Nodipecten subnodosus*) at Laguna Manuela, Mexico [29], the king scallop (*Pecten maximus*) from France and Norway, the Atlantic sea scallop (*Placopecten magellanicus*) from the Northwest Atlantic Ocean [30], the smooth scallop (*Flexopecten glaber*) from the Çardak Lagoon, Türkiye [31], the bay scallop (*Argopecten irradians*) in the North China Sea [32], and the Yesso scallop (*Mizuhopecten yessoensis*) in the Yellow Sea, China [33]. Substantial glycine concentration has also been detected in the muscle of the Mediterranean scallop (*Pecten jacobaeus*) from the Gulf of Antalya, Türkiye [34], as well as in the Zhikong scallop (*Chlamys farreri*) from the Yellow and Bohai Seas, China [35]. In the mantle of the Noble scallop (*Chlamys nobilis*) from the Taiwan Strait, China, glycine was prevalent in the amino acid composition [36]. Furthermore, elevated levels of glycine and arginine have been detected in protein isolates obtained from the gonads of the Yesso scallop (*Mizuhopecten yessoensis*) from the Yellow Sea, China [37].

The high content of glycine and arginine in scallop tissues can be attributed to their critical roles within scallop physiology. Glycine’s importance is highlighted by glycine-117, a residue found to be conserved across all sequenced molluscan regulatory light chains [38]. This particular residue is essential for hydrogen bond formation with the contiguous essential light chain, pivotal to the regulatory mechanism [39]. Moreover, the arginine phosphate present in scallops serves a similar metabolic function to phosphocreatine in vertebrates, although it is the former that facilitates the instantaneous generation of ATP required at the onset of muscle contraction. The unique anaerobic glycolysis pathway in scallops produces octopine instead of lactate, resulting from the reductive union of pyruvate and arginine [40]. This pathway is utilized both during environmental hypoxia and the functional hypoxia that occurs with high-intensity exercise [41].

Our findings revealed that arginine and leucine are less abundant in the mantle than in the muscle tissue. Analogous results were documented for *Chlamys nobilis* from Nan’ao Island, China [36]. In Iceland scallops, a high degree of dissimilarity between these two tissues was noted. The differences may be linked to the distinct structural and functional characteristics of these tissues. The mantle has significant roles in shell formation, gas and dissolved molecule exchange, as well as the secretion of enzymes, including acid phosphatase and phenoloxidase, which are involved in the structural alterations and tanning of the shell’s periostracal layer [28,42,43]. In contrast, the adductor muscle, which allows for rapid valve movements and is responsible for jet propulsion swimming, relies predominantly on anaerobic metabolism [44,45]. The difference in myosin types between the striated adductor muscle and other muscle types like the catch muscle, found also in the foot, mantle, gonad, and heart, may explain variances in amino acid content between muscles and mantle.

It was also noted that high contents of glycine and arginine in scallop muscles contribute to a sweet flavor that is generally preferred by consumers. The total daily intakes of essential amino acids in grams recommended by the World Health Organization for a 70 kg person calculated for the muscle of the Iceland scallop are as follows [46]: Thr 46, His 160, Val 192, Ile 302, Leu 460, Phe 468, and Lys 577. It is important to note that these values serve as a reference and are not meant to fulfill daily nutritional needs, considering the premium nature of the adductor muscle as a gourmet delicacy.

The outcomes of our PCA and SIMPER analyses indicated no significant variation in the amino acid composition among the three tissues examined. This finding suggests that scallop by-products such as gonads and mantles could be an excellent source for various applications in food processing, including the production of food additives, animal feed, and functional foods [47,48]. With the large volumes of marine processing by-products, there is a significant opportunity to develop valuable protein products from these resources. Recent research has explored the potential of scallop by-products, with studies revealing that proteins derived from yesso scallop gonads could form a novel functional matrix [37]. A study by Ri et al. [49] has shown that the major collagen in scallop mantles possesses a unique amino acid composition, paving the way for diverse applications, including the production of value-added functional foods. Likewise, Guo et al. [50] discovered that bay scallop proteins and their hydrolysates exhibit strong antioxidant activity and possess considerable nutritional value, offering potential for commercialization in the development of antioxidant-rich functional foods.

### 4.3. Factors

Statistical analyses revealed that amino acid contents in scallop muscles were inversely correlated with the GI, accounting for 19% of the observed variance. The process of gametogenesis is known to be highly energetic, requiring the translocation of nutrient reserves to the gonadal tissues to facilitate gamete development. Consequently, the observed negative correlation between muscle amino acid levels and the GI may be explained by the physiological reallocation of energy stores, mainly glycogen, from the muscle—which serves as a storage organ—to the gonadal tissue during the gametogenetic cycle [51]. This phenomenon exemplifies the bioenergetic trade-offs that occur when the energy allocation within an organism’s life history either closely matches or exceeds the net energy availability from the environment at any given time. In such scenarios, organisms face physiological trade-offs as multiple biological processes compete for limited energy reserves, requiring strategic allocation to ensure both survival and reproductive success [52].

Depth emerged as the second significant determinant for the amino acid composition of the adductor muscle, explaining an additional 16% of the variation and correlating positively with elevated amino acid content. This finding is likely attributable to varying food conditions at different depths. *Chlamys islandica*, a benthic suspension feeder, primarily consumes a diverse array of microalgae species in shallower habitats, whereas in deeper aquatic zones, its diet shifts towards detritus and suspended organic debris [53]. Detrital matter generally comprises more complex amino acid composition compared to phytoplankton, and often includes zooplankton remnants or organic detritus from benthic fauna, which are characterized by their attached bacterial communities known for their high enzymatic activity and amino acid content [54]. A study by Hao et al. [55] on yesso scallops near Zhangzi Island reported a similar positive relationship between depth and amino acid composition, with individuals from the bottom culture group demonstrating higher abundance of most amino acids compared to those from the suspended culture group.

The RDA did not reveal significant variations in amino acid contents in scallop gonads concerning the comprehensive set of factors considered in our study. This outcome may suggest that no significant physiological activity occurred in these organs during the study period, with the spawning of the current year being over and oogenesis just commencing [17].

Conversely, the RDA applied to mantle tissue data indicated that water temperature alone accounted for 26% of the variance in amino acid contents. Given the integral role of the mantle in both growth and enzyme secretion, particularly leucine aminopeptidase [28], and considering that these processes are accentuated at higher temperatures, the positive correlation between leucine levels and water temperature is in line with expectations. Similarly, a study by Beltrán-Lugo et al. [29] found increased leucine contents in *Nodipecten subnodosus* inhabiting warmer waters of Laguna Manuela, Mexico. In addition, Liu et al. [33] documented a temporal increase in glycine levels during the post-spawning phase that correlated with rising water temperatures in *Mizuhopecten yessoensis* from the Yellow Sea.

Experimental research suggests that a high fouling load and predators can modulate octopine levels, leading to significant alterations in arginine P levels [28]. The influence of biotic interactions on amino acid composition, as well as seasonal dynamics in amino acid composition, are topics of further studies.

## 5. Conclusions

Our study represents the first biochemical profiling of amino acids within the muscle, gonadal, and mantle tissues of Iceland scallops from the coastal Barents Sea. The highest contents were found for glycine and arginine, which is expected given the important roles of these amino acids in the metabolism of scallops. No significant differences were observed between male and female composition, and all three tissues exhibited insignificant dissimilarities from each other. For the first time, we conducted a multivariate analysis of the environmental factors in determining the amino acid composition of Iceland scallops from the Barents Sea. Our findings indicate no significant relationships for the gonad, most likely because our study was conducted after the main reproductive season. Meanwhile, the amino acid contents in the muscle were found to be influenced by the gonad index and depth, whereas in the case of the mantle, only water temperature showed a significant influence on the amino acid content. Our results highlight the promising potential of scallop by-products for the food and pharmaceutical industries and provide a valuable baseline for further research.

## Figures and Tables

**Table 1:** Biometric characteristics of Iceland scallops collected for amino acid analysis in Dalnezelenetskaya Bay, July 2022 and 2023.

Parameter	Male				Female				Total			
	Min	Max	X	SE	Min	Max	X	SE	Min	Max	X	SE
SL	76.5	105.7	90.1	3.8	72.2	96.0	85.4	3.0	72.2	105.7	87.6	2.4
TW	83.0	158.0	119.4	11.7	55.0	149.0	98.7	12.2	55.0	158.0	108.3	8.7
MW	2.0	6.7	4.9	0.7	1.2	23.9	5.9	3.0	1.2	23.9	5.4	1.6
GW	6.0	21.0	14.0	2.5	7.3	14.6	11.8	1.0	6.0	21.0	12.8	1.3
MW	6.3	17.0	11.1	1.5	4.2	9.8	7.4	0.9	4.2	17.0	9.1	1.0
GI	2.1	5.7	4.1	0.5	2.2	16.0	4.9	1.9	2.1	16.0	4.5	1.0
MI	6.5	14.6	11.3	1.2	8.5	14.3	12.4	0.8	6.5	14.6	11.8	0.7
MNI	7.6	10.8	9.2	0.6	6.5	8.3	7.5	0.2	6.5	10.8	8.3	0.4

Note. SL—shell length (mm), TW—total weight (g), MW—muscle weight (g), GW—gonad weight (g), MW—mantle weight (g), GI—gonad index (%), MI—Muscle index (%), MNI—mantle index (%), Min—minimum, Max—maximum, X—mean, SE—standard error.

**Table 2:** Amino acid content in the adductor muscle, gonad, and mantle of Iceland scallop from Dalnezelenetskaya Bay, Barents Sea.

Amino Acid	Muscle				Gonad				Mantle			
	Min	Max	X	SE	Min	Max	X	SE	Min	Max	X	SE
Aspartic acid	1.4	20.9	9.6	2.1	1.4	13.1	5.7	1.2	1.2	12.3	6.5	1.2
Glutamic acid	2.0	4.8	3.6	0.2	1.6	4.6	3.3	0.3	1.7	4.3	3.1	0.2
Hydroxyproline	0.3	0.9	0.6	0.1	0.3	1.1	0.7	0.1	0.3	3.7	0.8	0.2
Serine	1.1	6.8	3.5	0.6	1.3	7.7	3.6	0.6	1.4	18.5	4.6	1.2
Glycine	1.4	24.7	11.8	2.5	0.7	21.6	11.5	2.2	1.4	17.5	9.6	1.6
Histidine	0.8	15.1	4.4	1.2	0.7	10.6	2.8	0.7	1.2	8.7	3.2	0.6
Arginine	5.2	21.6	11.2	1.1	2.0	16.7	8.3	1.3	3.3	8.6	5.8	0.4
Threonine	1.1	16.3	6.0	1.3	1.1	9.4	4.6	0.7	1.4	9.8	4.7	0.8
Alanine	2.3	11.7	5.6	0.9	0.7	9.6	4.1	0.8	1.3	7.4	3.2	0.5
Proline	0.3	10.4	3.2	0.9	0.3	26.4	6.7	2.1	0.4	20.0	5.8	1.4
Tyrosine	1.3	8.3	4.0	0.5	1.6	7.9	3.9	0.6	1.1	5.4	2.9	0.4
Valine	1.2	34.4	9.5	2.8	1.1	19.6	6.7	1.8	1.5	18.5	5.8	1.5
Isoleucine	1.0	13.7	4.6	1.0	0.8	6.8	3.5	0.6	1.3	7.9	3.8	0.6
Leucine	1.8	9.8	5.9	0.8	1.0	9.0	4.3	0.7	1.2	6.9	3.3	0.5
Phenylalanine	0.4	10.7	3.7	0.9	0.4	7.4	2.3	0.6	0.6	4.9	2.5	0.4
Lysine	0.6	13.6	3.6	1.3	0.5	1.9	1.2	0.1	0.6	9.5	2.8	0.8

Note. Min—minimum, Max—maximum, X—mean, SE—standard error.

**Table 3:** Results of SIMPER analysis of amino acid data showing the amino acids that contributed the most to the dissimilarity between each scallop tissue sampled.

Amino Acid	Av.Diss	Diss/SD	Contrib%	Cum.%
	Muscle vs. Gonad, Dissimilarity 40.79%			
Glycine	6.06	1.35	14.87	14.87
Valine	5.44	0.87	13.35	28.22
Asparagine	4.33	1.37	10.62	38.84
Proline	3.52	0.88	8.64	47.48
Arginine	3.45	1.19	8.46	55.94
	Muscle vs. Mantle, Dissimilarity 41.10%			
Glycine	5.59	1.5	13.61	13.61
Valine	5.44	0.88	13.24	26.86
Asparagine	4.43	1.45	10.78	37.63
Arginine	3.67	1.52	8.92	46.55
Proline	2.92	1.14	7.1	53.66
	Gonad vs. Mantle, Dissimilarity 37.33%			
Glycine	5.9	1.43	15.79	15.79
Valine	4.37	0.86	11.7	27.49
Proline	4.35	1.03	11.65	39.14
Asparagine	3.3	1.32	8.84	47.98
Arginine	2.8	1.34	7.51	55.49

Note: Av.Diss—mean dissimilarity (%), Diss/SD—standard deviation, Contrib%—contribution to dissimilarity (%), Cum.%—cumulative contribution (%).

**Table 4:** List of environmental variables contributed to the RDA model based on amino acid contents in tissues of Iceland scallop from Dalnezelenetskaya Bay.

Variable	Muscle			Variable	Mantle		
	EV	F	p		EV	F	p
GI	19	2.64	0.009	T	26	4.71	0.002
D	16	2.69	0.041	SL	15	1.99	0.109
T	11	1.56	0.193	D	9	1.10	0.320
MI	8	1.34	0.233	MNI	6	1.03	0.365
MNI	5	0.87	0.488	GI	5	0.92	0.428
Sed	4	0.58	0.679	MI	7	1.43	0.230
SL	2	0.37	0.832	Sed	4	0.72	0.581

Note. D—depth, T—water temperature, SL—shell length, Sed—sediments, GI—gonad index, MI—muscle index, MNI—mantle index, EV—explained variation, %, F—pseudo F-ratio, p—probability level.

## Data Availability

Data are contained within the article.
